# Development of a Nationally Agreed Core Clinical Dataset for Childhood Onset Uveitis

**DOI:** 10.3389/fped.2022.881398

**Published:** 2022-06-21

**Authors:** Ameenat Lola Solebo, Salomey Kellett, Jugnoo Rahi, Reshma Pattani, Clive Edelsten, Andrew D. Dick, Alastair Denniston, The Pediatric Ocular Inflammation UNICORN Study Group, Ms Ameenat Lola Solebo

**Affiliations:** ^1^Population, Policy and Practice Programme, UCL GOS Institute of Child Health, London, United Kingdom; ^2^National Institute for Health Research Biomedical Research Center at UCL Great Ormond Street Institute of Child Health and Great Ormond Street Hospital, London, United Kingdom; ^3^Great Ormond Street Hospital for Children NHS Trust, London, United Kingdom; ^4^Ulverscroft Vision Research Group, Institute of Child Health, University College London, London, United Kingdom; ^5^National Institute for Health Research Biomedical Research Center at Moorfields Eye Hospital NHS Foundation Trust and UCL Institute of Ophthalmology, London, United Kingdom; ^6^Bristol Eye Hospital, University Hospitals Bristol NHS Foundation Trust, Bristol, United Kingdom; ^7^Translational Health Sciences, Faculty of Health Sciences, University of Bristol, Bristol, United Kingdom; ^8^University Hospitals Birmingham NHS Foundation Trust, Birmingham, United Kingdom; ^9^Academic Unit of Ophthalmology, Institute of Inflammation & Aging, College of Medical and Dental Sciences, University of Birmingham, Birmingham, United Kingdom

**Keywords:** uveitis, child, consensus, data collection, dataset, electronic health record

## Abstract

**Background:**

Childhood onset uveitis comprises a group of rare inflammatory disorders characterized by clinical heterogeneity, chronicity, and uncertainties around long term outcomes. Standardized, detailed datasets with harmonized clinical definitions and terminology are needed to enable the clinical research necessary to stratify disease phenotype and interrogate the putative determinants of health outcomes. We aimed to develop a core routine clinical collection dataset for clinicians managing children with uveitis, suitable for multicenter and national clinical and experimental research initiatives.

**Methods:**

Development of the dataset was undertaken in three phases: phase 1, a rapid review of published datasets used in clinical research studies; phase 2, a scoping review of disease or drug registries, national cohort studies and core outcome sets; and phase 3, a survey of members of a multicenter clinical network of specialists. Phases 1 and 2 provided candidates for a long list of variables for the dataset. In Phase 3, members of the UK's national network of stakeholder clinicians who manage childhood uveitis (the Pediatric Ocular Inflammation Group) were invited to select from this long-list their essential items for the core clinical dataset, to identify any omissions, and to support or revise the clinical definitions. Variables which met a threshold of at least 95% agreement were selected for inclusion in the core clinical dataset.

**Results:**

The reviews identified 42 relevant studies, and 9 disease or drug registries. In total, 138 discrete items were identified as candidates for the long-list. Of the 41 specialists invited to take part in the survey, 31 responded (response rate 78%). The survey resulted in inclusion of 89 data items within the final core dataset: 81 items to be collected at the first visit, and 64 items at follow up visits.

**Discussion:**

We report development of a novel consensus core clinical dataset for the routine collection of clinical data for children diagnosed with non-infectious uveitis. The development of the dataset will provide a standardized approach to data capture able to support observational clinical studies embedded within routine clinical care and electronic patient record capture. It will be validated through a national prospective cohort study, the Uveitis in childhood prospective national cohort study (UNICORNS).

## Introduction

Childhood uveitis, a group of inflammatory eye disorders characterized by overlapping clinical phenotypes, chronicity, and ongoing risk of visual morbidity, is a rare ‘disease' with an estimated incidence of up to 14 per 100,000 children (1–6). Between 10–25% of affected children reach adulthood having permanently lost some vision in at least one eye due to the complications which follow uncontrolled inflammation (1, 7, 8). Typically, disease activity continues into mid adulthood (9–11). The key to preventing uveitis-related blindness is prompt control of disease (1, 7, 8). Disease control is achieved using a combination of topical and systemic corticosteroids, systemic disease modifying immunomodulatory agents, and biologic therapies (12). There are several areas of uncertainty including etiology, disease subtype, disease burden (with studies reporting a 20-fold difference in incidence rates) (1–6), and uncertainties around the predictors of disease outcome (1–6). Several large but methodologically heterogenous studies have reached differing conclusions on the predictors of ocular outcome or therapeutic response, including conflicting findings on gender (13–15), the role of topical steroids (16, 17) and age at onset, analysis of which is typically confounded by disease duration and ANA status (8, 18–21). There is a need for research evidence to inform health policy and clinical practice, particularly for non-JIA associated disease, by identifying the predictors and mediators of disease risk, and of poor disease outcome.

Clinical research on rare disorders, such as childhood uveitis, faces the challenge typical to all rare disease research: reaching sufficiently large study sizes to allow robust statistical analysis. This challenge is usually addressed by undertaking multicenter studies, whose success relies not only on increased sample size, but also on standardized collection of the granular data necessary to interrogate variables of interest. We aimed to develop a core clinical dataset for specialists managing children with uveitis, to facilitate routine standardized data collection.

## Methods

We undertook a three phase process to develop the core dataset. The phases comprised:

### Phase 1: A Rapid Review of the Evidence Base

We undertook a rapid scoping review (22) of the data collection undertaken in studies of disease outcome in childhood uveitides. The aim was to inform the development of a provisional list of variables to be collected, and to describe the definitions used for those variables, by mapping the literature and identifying key concepts. A Pubmed search was undertaken on 2nd April 2018 using the terms (uveitis) AND (cohort study) AND ((infant[MeSH] OR child[MeSH] OR adolescent[MeSH])). Identified studies were eligible for inclusion if they were (i) prospective (ii) involved children (aged under 18 years) with uveitis. We also included retrospective consecutive case series of children with uveitis with sample sizes >20 children. Ineligible studies were those limited to infectious uveitis. The data extracted from each study comprised the data collected by the investigators for that study, and any validated definitions used for clinical variables of interest. As this was a rapid review, a single reviewer (ALS) undertook the search, study selection and data extraction.

### Phase 2: Collation of National and International Datasets and Core Outcome Sets for Children With Juvenile Idiopathic Arthritis With and/or Without Associated Uveitis

In order to further inform the long-list of potential variables, we sought to identify any protocols or core outcome sets for national and international collaborative studies which involved outcomes for the population of children who typically make up the largest single sub-group in studies of childhood uveitis: those with JIA. The protocols for the national or international prospective studies or registries for JIA were identified via national or multinational pediatric rheumatological groups, specifically the Childhood Arthritis and Rheumatology Research Alliance (CARRA, USA), British Society for Pediatric and Adolescent Rheumatology (BSPAR, UK), the Danish and German Pediatric Rheumatologist Networks, and the rheumatology led multinational interdisciplinary working group for uveitis in childhood (MIWGUC) (23). We also sought to identify any nation-level datasets or outcome sets through the UK's Royal College of Ophthalmologists.

### Phase 3: Consensus Exercise

A network of clinicians (consultant ophthalmologists and rheumatologists) who manage childhood uveitis were invited to take part in an exercise to rationalize and agree on the list of variables which would form the core clinical dataset. Eligible clinicians were those consultants (‘attending' clinicians) who managed children with uveitis, or managed young adults with childhood onset uveitis, within UK specialist care centers. Clinicians were identified through their membership of the UK's Pediatric Ocular Inflammation Group, POIG (24). Participating clinicians were approached electronically between May 2019 and November 2019 and asked to independently review a Microsoft Excel worksheet containing the long-list. They were asked to select from the long-list of variables their own minimal necessary dataset for collection in routine clinical practice (at the initial ‘new patient' consultation and at follow-up visits), to agree on the format for collection, and to identify any omissions. Consultants were also asked whether they agreed with the definitions of clinical terminology. Definitions were provided using embedded links within the worksheet. They were also invited to comment on the variables (e.g., the variable outcome choices) within the list. Variables were accepted onto the core dataset if more than 95% of clinicians agreed to routine collection. This threshold was agreed by the core research group, with the understanding that any failure of a long-list variable to reach this agreement threshold would not prevent the ongoing collection of that variable by the individual clinical teams.

This study received the necessary approvals from the relevant institutional research bodies (UK Health Research Authority Research Ethics Committee, IRAS 258638, REC 20/LO/0661). Patients were not involved in the undertaking of this study which sought to capture and report the minimal core dataset routinely collected by clinicians managing disease, but in a 2013 James Lind Alliance Priority Setting Partnership exercise, patients and professionals had prioritized outcomes for research for ocular inflammatory disease.

## Results

### Phase 1

The rapid review identified 2092 individual titles and abstracts. Screening of these abstracts resulted in the selection of 167 potentially eligible studies for full text review. Following full text review, there were 42 eligible studies (Supplemental Document 1), of which five studies were prospective (25–29). Clinical variables collected by these studies were collated in order to populate the long-list, with a total of 98 discrete data items, of which 8 were data items which were necessary for provision of clinical care and patient follow up (patient identifiers including hospital number, name and date of birth).

### Phase 2

The review of existing datasets used in national and multi-national pediatric rheumatology and Uveitis studies and registries resulted in the identification of five cohort studies (four for JIA, one for Uveitis; four national, one multinational, [Supplemental Document 2]), five drug registries (four for children treated with biologics for rheumatological disease, and one for adults treated with systemic immunosuppression for ocular inflammatory disease) and four disease registries (all for juvenile idiopathic arthritis). This resulted in an additional 18 discrete data items (Supplemental Document 3) on extraocular disease assessment, including family and medical history, broad assessment of systemic health and systemic disease activity, and involvement of other child health specialists. We also identified, through the UK's Royal College of Ophthalmologists, a recommended dataset for clinical care for adult inflammatory disease (30), and through the POIG, a previously published Delphi consensus generated list of baseline diagnostic investigations for childhood ocular inflammatory disease (31). This led to the addition of 16 items on diagnostic testing, and three on imaging, as well as informing the definition of variables on disease type and clinical findings. The resultant long-list comprised 138 items (Supplemental Document 3).

### Phase 3

We identified 41 clinicians who provide clinical care to children with inflammatory eye disease; 34 provided care to children only, with seven providing care to children and adults. Following approach of all those identified, responses were received from 31 (response rate 78%) comprising rheumatologists *n* = 1, ophthalmologists n = 28, and ophthalmologist/clinical nurse specialist multi-disciplinary teams (*n* = 2). No new data items were suggested.

In addition to the 8 necessary data items (patient identifiers such as name and hospital number), 89 data items were accepted by respondents for the final core clinical dataset (Table 1), 81 items to be collected at the first clinic appointment, and 64 items at follow up visits. The items listed in table 1 are provided to indicate the content, if not the exact format in which the question would be asked or captured.

**Table 1:** Core clinical dataset for childhood uveitis.

**Data item**	**Format of data entry**	**First Clinic appt**	**Follow up**
***Patient details***			
*Hospital number*	Alphanumeric	**X**	
*NHS number*	Number	**X**	
*Surname*	Free text	**X**	
*Forename*	Free text	**X**	
*DOB*	DD/MM/YYYY	**X**	
*Gender*	Male / female / other	**X**	
*Ethnicity*	UK Office of National Statistics classification system	**X**	
*Postcode*	Free text	**X**	
***Referral pathway***			
*Mode of detection*	Select one from: school screening / routine testing / routine surveillance / symptoms or concerns / other; if other, please specify [free text] - free text	**X**	
*Date problem started / detected*	MM/YYYY	**X**	
*Referral source*	Select one from: GP / rheumatologist / optician / school screening / A&E / secondary care / tertiary care / other; if other, please specify [free text]	**X**	
*Symptoms*	Select one or more from none / unsure / no details / blurred vision / redness / pain / discomfort / change in eye appearance / unknown / other; if other, please specify [free text] - free text	**X**	**X**
*Other history*	Free text	**X**	
*Other referral details*	Free text	**X**	
*Date first seen*	DD/MM/YYYY	**X**	
***Previous uveitis details (if diagnosed prior to first clinic appointment)***			
*Date uveitis first diagnosed*	DD/MM/YYYY	**X**	
*Date topical treatment started*	DD/MM/YYYY	**X**	
*Previous uveitis event*	Select one or more from: none / surgery / treatment started / treatment stopped / increased IOP / VA loss / other; if other, please specify [free text] - free text	**X**	
*Date previous uveitis event*	DD/MM/YYYY	**X**	
*Details*	Free text	**X**	
***Systemic disease / disorders***			
*Newly diagnosed / previously known systemic diagnoses*	Select one from: none / JIA (inc ILAR subtype) / definite sarcoid / presumed sarcoid / probable sarcoid (32) / blau / behcets / tinu / psoriasis / IBD / vasculitis / other; if other, please specify [free text]	**X**	**X**
*If JIA, subtype*	Select one from systemic / oligo / poly / psoriatic / ERA / undifferentiated	**X**	**X**
*If vasculitis, subtype*	Free text	**X**	**X**
*Date onset of systemic disease*	DD/MM/YYYY	**X**	
*Details systemic diagnosis*	Free text	**X**	
*Systemic review (where diagnosis unknown, or undifferentiated JIA)*	Select one or more from: no concerns / fever / rash or spots / weight change / lymphadenopathy / lower GI symptoms / oral or other upper gi symptoms / respiratory symptoms / other; if any concerns, please specify [free text]	**X**	**X**
*Family history*	All that apply from: no relevant history / unknown / glaucoma / uveitis (type) / JIA / spondylopathy / RA / sarcoid / SLE / TB / MS / coeliac / thyroid / IBD / other autoimmune disease / other; if other, please specify [free text]	**X**	
*Details family history*	Free text	**X**	
***Previous medication***			
*Previous / current ocular meds*	Select one from: predforte / dexamethasone / cyclopentolate / other; if other, please specify [free text]	**X**	
*Date started*	DD/MM/YYYY	**X**	
*Date stopped (if no longer in use)*	DD/MM/YYYY	**X**	
*Previous / current systemic medication*	Free text	**X**	
*Date previous systemic medication started*	DD/MM/YYYY	**X**	
*Date previous systemic medication stopped (if no longer in use)*	DD/MM/YYYY	**X**	
***Eye examination***			
*Date (if different to Date first seen)*	DD/MM/YYYY	**X**	**X**
*Chart used*	Select one from: standard LogMAR / kays pictures / cardiff cards / snellen / other; if other, please specify [free text]	**X**	**X**
*VA tested with*	Select one from unaided / aided / pinhole	**X**	**X**
*VA recorded*	With quantitative or qualitative units, ie: LogMAR / snellen / CPD / CF / HM / PL / NPL	**X**	**X**
*Eye tested*	Recorded for each of RE / LE / where necessary BEO	**X**	**X**
Any change to anterior segment^*^	Yes / No		**X**
Any change to posterior segment^*^	Yes / No		**X**
AC cells pre dilation^*^	Limited to 0 / 0.5+ / 1+ / 2+ / 3+ / 4+ [definition in (33) and (30)]	**X**	**X**
AC flare pre dilation^*^	Limited to 0 / 0.5+ / 1+ / 2+ / 3+ / 4+ [definition in (33) and (30)]	**X**	**X**
Keratic precipitates^*^	No / Yes + descriptor	**X**	**X**
Lens^*^	clear / cataract / aphakic / pseudophakic / other; if other, please specify [free text]	**X**	**X**
If cataract: predominant cataract type^*^	Limited to: total / cortical / anterior / posterior / nuclear	**X**	**X**
Band keratopathy^*^	No / Yes peripheral / Yes central axis	**X**	**X**
Pupillary synechiae^*^	No / Yes, please specify (in clock h)	**X**	**X**
Iris bombe^*^	Yes / No	**X**	**X**
Other iris abnormality details^*^	None / PAS (in clock h) / Nodules / Atrophy / Other; if Other, please specify [free text]	**X**	**X**
Pupillary membrane formation^*^	Yes / No	**X**	**X**
Other anterior segment^*^	Selection from: none / Scleritis / Keratitis / Iris atrophy / Other; if Other, please specify [free text]	**X**	**X**
Dilation^*^	Yes / No	**X**	**X**
Vitreous haze^*^	Limited to: 0/0.5+/1+/2+/3+/4+ [definition in (33) and (30)]	**X**	**X**
Posterior segment healthy^*^	Yes / No / No view	**X**	**X**
Vitreous cells^*^	Yes / No	**X**	**X**
Vitreous opacities^*^	Select one from: none / exudate over pars plana or snowbanking / snowballs / other; if other, please specify [free text]	**X**	**X**
CD ratio^*^	0–1 Decimal	**X**	**X**
Disc swelling^*^	Yes / No	**X**	**X**
Other disc changes^*^	All that apply from: none / Disc hemorrhages / Hyperaemia / Vessel engorgement / Vessel obscuration / Other; if Other, please specify [free text]	**X**	**X**
Optic atrophy^*^	Yes / No	**X**	**X**
Macula oedema present^*^	No / Yes-clinical / Yes-OCT	**X**	**X**
ERM^*^	Yes / No	**X**	**X**
Retinal vasculitis^*^	Yes / No	**X**	**X**
Any active chorioretinal lesion^*^	No / peripheral only / macula	**X**	**X**
Other posterior segment^*^	All that apply from: none / retinal neovascularisation / CNV / subretinal fluid / subretinal mass / vitreous hemorrhage / other structural macula change / retinal detachment / other; if other, please specify [free text]	**X**	**X**
IOP (mmHg)^*^	Numerical	**X**	**X**
Test used IOP^*^	Goldmann / I-care / Digital	**X**	**X**
Glaucoma^*^	Yes / No (defined ocular hypertension PLUS sign(s) of raised IOP – corneal changes, axial length increase or myopic shift, optic disc change, or visual field loss)	**X**	**X**
***Disease summary***			
*Type of uveitis*	Limited to anterior / anterior with intermediate / intermediate / posterior / panuveitis (30)	**X**	**X**
*Subtype of uveitis*	Limited to: iritis / iridocyclitis / anterior cyclitis / pars planitis / posterior cyclitis / hyalitis / focal, multifocal, or diffuse choroiditis / chorioretinitis / retinochoroiditis / retinitis / neuroretinitis [definition in (33) and (30)]	**X**	**X**
*Cause(s) of reduced VA*	All that apply of: cataract / refractive error / vitreous / cmo / amblyopia / AC inflammation / cornea / CNV / other; if other, please specify [free text]	**X**	**X**
***Topical treatment***			
Topical corticosteroid drop^*^	Select one from: none / dexamethasone / predforte / lotemax / maxitrol / fml / other; if other, please specify [free text]	**X**	**X**
Steroid drops daily frequency^*^	Select one from: alt day / 1 / 2 / 3 / 4 / 6 / 2 hourly / hourly / other; if other, please specify [free text]	**X**	**X**
Glaucoma drops^*^	Select from: none / timolol / dorzolamide / brinzolamide / brimonidine / iopidine / latanoprost / bimatoprost / travoprost / other; if other, please specify [free text]	**X**	**X**
Mydriatic drops^*^	select one from: none / cyclopentolate / tropicamide / atropine / other; if other, please specify [free text]	**X**	**X**
Mydriatic drop daily use frequency^*^	select one from: alt days / 1 / 2 / 3 / 4	**X**	**X**
***Systemic treatment***			
*New / changed systemic treatment*	Yes / No	**X**	**X**
*Drug / route / dose*	Free text, or select from prednisolone / methotrexate SC / methotrexate PO / mycophenolate mofetil / adalimumab / infliximab / tocilizumab SC / tocilizumab IV / acetazolamide PO /	**X**	**X**
*Date started*	DD/MM/YYYY (for each individual treatment)	**X**	**X**
*Date stopped*	DD/MM/YYYY (for each individual treatment)	**X**	**X**
***Investigations***			
*Fundal photography*	Not done / done + normal / done + abnormal, please specify [free text]	**X**	**X**
OCT macula^*^	Not done / done + normal+cmt / done + abnormal findings+CMT, please specify [free text]	**X**	**X**
OCT optic nerve^*^	Not done / done + normal / done + abnormal, please specify [free text]	**X**	**X**
*ANA*	Not done / done, with titer	**X**	
*HLA-B27*	Not done / done, negative result / done, positive result	**X**	
*FBC*	Not done / done, normal / done, abnormal, please specify [free text]	**X**	
*Other notable positive laboratory findings*	No / Yes, - please specify [free text]	**X**	**X**
***Other management***			
*Referral to pediatrician*	Yes / No	**X**	**X**
*Other referral*	Free text	**X**	**X**
*Date of any surgery*	DD/MM/YYYY	**X**	**X**
***Surgical treatment***			
Indication for surgery^*^	Select one from: intractable inflammation / iris bombe / cataract / glaucoma / BK / RD / ERM / Other; if other, please specify [free text]	**X**	**X**
Type of surgery^*^	Select from: intraocular steroid/periocular steroid / lens extraction / IOL / YAG capsulotomy / Surgical caps + Vitrectomy / PI / RD surgery / Dexamethasone implant (Ozurdex) / Intravitreal anti-VEGF / Vitrectomy / Glaucoma shunt surgery / Glaucoma trabeculectomy / Glaucoma cyclodiode / Removal BK / Other; if Other, please specify [free text]		**X**
*Peri-operative steroid pulse*	No / Yes, please specify [free text]		**X**
*Other relevant surgical details*	Free text		**X**
*Complications following surgery*	No / Yes, please specify [free text]		**X**

* *Reported for each eye separately. GP, General practitioner / primary care physician; A&E, accident and emergency; IOP, intraocular pressure; VA, Visual acuity; JIA, Juvenile Idiopathic Arthritis; ILAR, International League of Associations for Rheumatology; TINU, Tubulointerstitial Nephritis and Uveitis Syndrome; IBD, Inflammatory bowel disease; ERA, enthesitis related arthritis; GI, gastrointestinal; RA, Rheumatoid arthritis; SLE, Systemic lupus erythematosus; TB, tuberculosis; MS, Multiple sclerosis; CPD, cycles per degree; CF counting fingers; HM, hand movements; PL, perception of light; RE right eye; LE, left eye, BEO, both eyes open; AC, anterior chamber; PAS, peripheral anterior synechiae; CMO, cystoid macular oedema; CNV, choroidal neovascular membrane; alt, alternate; CDR, cup disk ratio; OCT, optical coherence tomography; FBC, full blood count; HLA, human leukocyte antigen; CMT, central macular thickness; BK, band keratopathy; RD, retinal detachment; ERM, epiretinal membrane; IOL, intraocular lens; PI, Peripheral iridectomy; VEGF, Vascular endothelial growth factor*.

#### Referral Pathway

Respondents agreed to the collection of data items on the mode of detection of ocular inflammation, and on the referral pathway of the child into the specialist clinic or center. There was also agreement on the collection of information on symptomatology.

#### Presence and Status of Systemic Disorders

Although respondents agreed to collect information on the presence of associated systemic disease or on symptoms indicative of systemic inflammatory disease, there was no agreement on inclusion of scores of non-ophthalmic disease activity within the dataset (27/31 agreed, 87%), with some clinicians remarking that they were “unsure as to whether rheumatologists collect(ed) this information” or that this data collection “seems to be primarily the job of rheumatology team”. There was also no agreement on routine measurement of height and weight (28/31 agreed, 90%). The only serological investigations which were agreed for inclusion were full blood count, anti-nuclear antibody, and HLA B27. The investigations which were not included were however typically collected by the majority of respondents: chest X-ray, quantiferon/Interferon-Gamma Release Assays, Rheumatoid factor, erythrocyte sedimentation rate, c-reactive protein, angiotensin converting enzyme, liver and renal function testing or investigation status routinely recorded by 29/31, 94%; antineutrophil cytoplasmic antibody, anticentromere antibody, antistreptolysin O titer and vitamin D level, 28/31 or 90%; immunoglobulin levels, 27/31 or 87%; routine recording of testing for mutations in the NOD2 gene was undertaken by 14/31 or 45%.

#### Ophthalmic Assessment

Almost all the data items referring to clinical examination were agreed for inclusion. Excluded items comprised routine assessment for strabismus (26/31, 84%), color visual function (26/31, 84%), near vision (27/31, 87%) or contrast acuity (13/31, 42%).

Only 6 of the 31 responding clinicians agreed to inclusion of laser flare photometry, with the majority of respondents stating that they did not have access to a photometer in clinic. Documentation of fundus dye-based angiography investigations were also not included within the core dataset.

## Conclusion

We report an expert consensus core clinical dataset for the routine collection of clinical data for children diagnosed with non-infectious uveitis, including data items on demographic details, mode of detection, ocular disease phenotype, ocular disease activity and severity, co-existent systemic disease, management, and laboratory and imaging findings. Although this has been anchored in clinical practice across centers throughout one particular country (UK), the standardization provided through the dataset may be helpful in supporting multicenter research collaboration not only at national, but potentially at international level. Many clinical variables on the ‘long-list' which were not included in the core dataset will still be collected by the majority of centers, supporting ongoing development of the dataset.

The core clinical dataset developed here was based on a three-phase approach which culminated in a single survey of clinician experts resulting in selection based on pre-defined inclusion threshold. This work was designed to serve as a baseline tool for further exploration and validation in a prospective study (Uveitis in Childhood National Prospective Cohort, UNICORN study) in which multistakeholder input will be gathered, and in which the tool could be revised as needed. Our approach to the development of this core clinical dataset is aligned to other work in this field (34–36). Our work represents a key step toward this future validated, multi-stakeholder consensus core dataset including providing the ‘long list', definitions and data structure. Consensus based clinical datasets are strengthened through regular review and updates, and this work has created the infrastructure necessary for that activity at a national level. Only one pediatric rheumatologist was involved at this stage, with resultant possible omission of key extra-ophthalmic variables, despite the review including pediatric rheumatology evidence. Future work will involve a broader panel of child health specialists. This study was strengthened by the support of a multicenter group of specialists who represented practice across the four member states of the United Kingdom (England, Scotland, Northern Ireland and Wales). The striking level of consensus across the network is reflective of the collaborative nature of the group, who have previously undertaken a Delphi exercise and surveys on childhood inflammatory eye disease (31, 37).

There are a number of interesting and potentially important omissions in our core clinical dataset, that future work is likely to address. First, the process did not identify and include any patient reported outcome measures (PROMs). Our work and that of others has highlighted the importance of a child-centered approach to clinical care, including the field of eye health, and the supporting power of PROMs. Childhood uveitis confers significant risk of psychosocial morbidity due to the impact of chronicity, visual impairment, treatment related adverse experiences, and the uncertainties of outcome and treatment choice inherent to these rare and complex diseases (38). It may be that a metric as simple as a single patient reported visual analog score may be sufficient to reliably and repeatably capture a child's or family's perceptions of disease state. However, internationally, there is a paucity of appropriate validated tools for use as PROMs in childhood uveitis (39). Quality of life metrics are particularly important in uveitis, with the burden conferred by the use of multiple systemic therapies. A recently validated tool for use in North American populations (the EYE-Q) has been shown to reliably, repeatably and responsively capture patient centered outcomes (quality of life) in childhood uveitis (40, 41), but this tool has not yet been translated for a British setting or validated for use in British populations. Such work (validation of the translation) as well as the increasing routine capture of patient reported outcomes in clinical practice will lead to an update in the clinical dataset presented here.

Objective metrics of anterior chamber inflammation are absent from this dataset. Anterior uveitis is the most common form of disease in childhood (6, 42), and currently, the only validated objective metric of disease state for anterior uveitis is laser flare photometry (LFP) (43). Our findings of low adoption of LFP across the UK is consistent with other evidence of poor uptake of the technology internationally (44). Anterior segment optical coherence tomography (AS-OCT) scanning may, in future, provide validated objective metrics of disease activity (45, 46), with AS-OCT being another potential addition in an updated dataset.

In recognition of the need to provide a “standardized language” for clinical care, outcome analysis, clinical audit and research (30), the UK's Royal College of Ophthalmologists has developed a recommended minimum mandatory dataset for clinical data collection in the management of uveitis (30). This mandatory dataset covers the definition of the type of uveitis (according to anatomical classification, onset, course and etiology), the severity of disease, the major therapeutic interventions and current disease status including logMAR acuity. Whilst the work presented here was informed by that dataset, there was a clear consensus about the need to build on this dataset in order to generate more detailed data, and to make it suitable for pediatric practice. For example, the categorization of disease as recurrent and chronic, or as sudden versus insidious, may not be possible for many children with asymptomatic anterior uveitis. Measures of function such as visual field testing and Snellen acuity may not be appropriate for younger children because of their inability to comply with such tests. There was also a consensus amongst survey responders on the need for standardized documentation of non-ophthalmic features, which have not yet been adopted in the RCOphth uveitis dataset.

Although our core clinical dataset is not designed as an outcome set, it is useful to review the items selected for inclusion within our dataset in the context of any published or proposed core outcome sets relevant to the field of childhood uveitis. One such outcome set undergoing development is the JIA associated uveitis outcome measure set from the Multinational Interdisciplinary Working Group for Uveitis in Childhood (MIWGUC) (23). Outside of the use of PROMS, there is strong alignment with the core clinical dataset presented here, which can be used to populate an outcome set such as that proposed by MIWGUC, and can support the interrogation of which factors determine these clinically important outcomes.

The wider adoption of a variety of electronic patient record systems across different health care settings brings opportunities to standardize the ‘real world' data being collected across these systems. A standardized language will underpin the development and optimization of electronic patient records, and supports ‘FAIR' (Findable, Accessible, Interoperable Reusable) clinical data. Future validation of this consensus-based clinical dataset and the harmonization of multi-center data collection will be provided by the currently underway UNICORN study, a national prospective inception cohort of children (aged <18yrs) newly diagnosed with non-infectious uveitis, with clinical, sociodemographic and patient reported data collection, enabling investigation of possible disease determinants, and the interrogation of patient centered outcomes. The work presented here should support the creation of research-ready datasets from routinely collected data, for use in the UNICORN study but also for use in future studies of clinical care, natural history, and outcomes.

## Data Availability Statement

The datasets presented in this study can be found in online repositories. The names of the repository/repositories and accession number(s) can be found in the article/Supplementary Material.

## Pediatric Ocular Inflammation Unicorn Study Group

Ms Ameenat Lola Solebo, NIHR Clinician Scientist and Consultant Ophthalmologist, UCL GOS Institute of Child Health, London, UK; Ms Salomey Kellett, Researcher, UCL GOS Institute of Child Health, London, UK; Professor Andrew D Dick, Professor of Translational Health Sciences, Bristol Medical School, University of Bristol, Bristol, UK; Professor Jugnoo Rahi, Professor of Ophthalmic Epidemiology, UCL GOS Institute of Child Health, London, UK; Professor Alastair Denniston, Professor of Ocular Inflammation, University of Birmingham, UK; Mr Damien C.M.Yeo, Consultant Ophthalmologist, Alder Hey Children's NHS Foundation Trust, Liverpool; Mr Jose Gonzalez-Martin, Consultant Ophthalmologist, Alder Hey Children's NHS Foundation Trust, Liverpool; Ms Eibhlin McLoone, Consultant Ophthalmologist, Belfast Royal Victoria Hospital NHS Foundation Trust; Prof Rachel Pilling, Consultant Ophthalmologist, School of Optometry and Vision Science, University of Bradford; Mr John Bradbury, Consultant Ophthalmologist, Bradford Teaching Hospitals NHS Foundation Trust, Bradford Royal Infirmary; Prof Athimalaipet V Ramanan, Consultant Pediatric Rheumatologist, University Hospitals Bristol NHS Foundation Trust; Ms Catherine Guly, Consultant Ophthalmologist, University Hospitals Bristol NHS Foundation Trust; Ms Brinda Muthusamy, Consultant Ophthalmologist, Cambridge University Hospital NHS Foundation Trust; Mr Patrick Watts, Consultant Ophthalmologist, Cardiff and Vale University Health Board Hospitals; Ms Christine Twomey, Clinical Nurse Specialist, Great Ormond Street Hospital NHS Foundation Trust; Ms Reshma Pattani, Specialist Optometrist, Great Ormond Street Hospital NHS Foundation Trust; Mr Clive Edelsten, Consultant Ophthalmologist, Great Ormond Street Hospital NHS Foundation Trust; Mr Daniel Pharoah, Consultant Ophthalmologist, James Paget University Hospitals NHS Foundation Trust; Mr Vernon Long, Leeds Teaching Hospitals NHS Trust, St. James's University Hospital; Mr Adam Bates, Consultant Ophthalmologist, Maidstone and Tunbridge Wells NHS Trust; Ms Elisabetta Scoppettuolo, Consultant Ophthalmologist, Maidstone and Tunbridge Wells NHS Trust; Prof Jane Ashworth, Consultant Ophthalmologist, Manchester University NHS Foundation Trust; Ms Laura Steeples, Consultant Ophthalmologist, Manchester University NHS Foundation Trust; Mr Harry Petrushkin, Consultant Ophthalmologist, Moorfields Eye Hospital NHS Foundation Trust and Great Ormond Street Hospital NHS Foundation Trust; Ms Dhanes Thomas, Consultant Ophthalmologist, Moorfields Eye Hospital NHS Foundation Trust; Mr Alan John Connor, Consultant Ophthalmologist, Newcastle Upon Tyne Hospitals NHS Foundation Trust; Dr Una O'Colmain, Consultant Ophthalmologist, Ninewells Hospital, Tayside Scotland NHS Board; Mr Anas Injarie, Consultant Ophthalmologist, Norfolk and Norwich University Hospitals NHS Foundation Trust; Mr Narman Puvanachandra, Consultant Ophthalmologist, Norfolk and Norwich University Hospitals NHS Foundation Trust; Ms Archana Pradeep, Consultant Ophthalmologist, Nottingham University Hospitals NHS Trust; Ms Srilakshmi Sharma, Consultant Ophthalmologist, Oxford University Hospitals NHS Foundation Trust; Dr Conrad Schmoll, Consultant Ophthalmologist, Princess Alexandra Eye Pavilion / Royal Hospital for Children and Young People, Edinburgh, Lothian NHS Board; Dr Eoghan Millar, Consultant Ophthalmologist, Royal Children's Hospital, NHS Greater Glasgow and Clyde; Mrs Kate Bush, Consultant Ophthalmologist, Royal Bournemouth Christchurch Hospitals NHS Foundation Trust; Mr M. Ashwin Reddy, Consultant Ophthalmologist, Royal London Hospital, Barts Health NHS Trust; Ms Jessy Choi, Consultant Ophthalmologist, Sheffield Children's Hospitals NHS Foundation Trust, and Sheffield Teaching Hospitals NHS Foundation Trust; Ms Gisella Cooper, Clinical Nurse Specialist, Sheffield Children's Hospitals NHS Foundation Trust, and Sheffield Teaching Hospitals NHS Foundation Trust; Ms Kristina May, Consultant Ophthalmologist, University Hospital Southampton NHS Foundation Trust, Southampton General Hospital; Mr Ed Hughes, Consultant Ophthalmologist, Sussex Eye Hospital, University Hospitals Sussex NHS Foundation Trust; Ms Ailsa Ritchie, Consultant Ophthalmologist, St Thomas' Hospital, Guys and St Thomas' NHS Trust

## Author Contributions

AS conceptualized, designed the study, and drafted the manuscript. AS, JR, ADD, AD, RP, and CE contributed to study design. AS and SK undertook analyses. All authors contributed to data collection. All authors critically reviewed the manuscript. All authors contributed to the article and approved the submitted version.

## Funding

AS and SK are supported by an NIHR Clinician Scientist award CS-2018-18-ST2-005). This work is part supported by a Wellcome Grant 204841/Z/16/Z. JR is supported in part by the NIHR BRC based at Moorfields Eye Hospital NHS Foundation Trust and UCL Institute of Ophthalmology, and an NIHR Senior Investigator award. This work was undertaken at UCL Institute of Child Health/Great Ormond Street Hospital for children which received a proportion of funding from the Department of Health's NIHR Biomedical Research Centers funding scheme.

## Conflict of Interest

The authors declare that the research was conducted in the absence of any commercial or financial relationships that could be construed as a potential conflict of interest.

## Publisher's Note

All claims expressed in this article are solely those of the authors and do not necessarily represent those of their affiliated organizations, or those of the publisher, the editors and the reviewers. Any product that may be evaluated in this article, or claim that may be made by its manufacturer, is not guaranteed or endorsed by the publisher.

## Supplementary Material

Supplementary Document 1 List of studies used to inform the long-list in Phase 1.

Supplementary Document 2 List of cohorts, registries and core outcome sets used to inform the long-list in Phase 1.

Supplementary Document 3 Long list of candidate items.
