# Supplementary material for: Development of a Nationally Agreed Core Clinical Dataset for Childhood Onset Uveitis
Source: Front Pediatr. 2022 Jun 21;10:881398. doi: 10.3389/fped.2022.881398 (PMC9253543; doi:10.3389/fped.2022.881398)

Supplementary document 1: List of studies used to inform the long-list in Phase 1

Title	Authors	Pub Year	Journal	Vol	Iss	Pages
Visual outcomes prognosticators in juvenile rheumatoid arthritis-associated uveitis	Dana MR; Merayo-Lloves J; Schaumberg DA; Foster CS	1997	Ophthalmology	104	2	236-44
Pars planitis: epidemiology, treatment, and association with multiple sclerosis.	Prieto JF; Dios E; Gutierrez JM; Mayo A; Calonge M; Herreras JM	2001	Ocul Immunol Inflamm	9	2	93-102
Occurrence of uveitis in recently diagnosed juvenile chronic arthritis: a prospective study.	Kotaniemi K; Kautiainen H; Karma A; Aho K	2001	Ophthalmology	108	11	2071-5
An evaluation of baseline risk factors predicting severity in juvenile idiopathic arthritis associated uveitis and other chronic anterior uveitis in early childhood.	Edelsten C; Lee V; Bentley CR; Kanski JJ; Graham EM	2002	Br J Ophthalmol	86	1	51-6
Autoimmune uveitis in children: clinical correlation between antinuclear antibody positivity and ocular recurrences.	Manzotti F; Orsoni JG; Zavota L; Cimino L; Zola E; Bonaguri C	2002	Rheumatol Int	21	4	127-32
Early predictors of severe course of uveitis in oligoarticular juvenile idiopathic arthritis.	Zulian F; Martini G; Falcini F; Gerloni V; Zannin ME; Pinello L; Fantini F; Facchin P	2002	J Rheumatol	29	11	2446-53
Long-term follow-up of 246 adults with juvenile idiopathic arthritis: functional outcome.	Packham JC; Hall MA	2002	Rheumatology (Oxford)	41	12	1428-35
Visual loss in uveitis of childhood.	de Boer J; Wulffraat N; Rothova A	2003	Br J Ophthalmol	87	7	879-84
Visual loss associated with pediatric uveitis in english primary and referral centers.	Edelsten C; Reddy MA; Stanford MR; Graham EM	2003	Am J Ophthalmol	135	5	676-80
Prognosis of juvenile rheumatoid arthritis-associated uveitis.	Paroli MP; Speranza S; Marino M; Pirraglia MP; Pivetti-Pezzi P	2003	Eur J Ophthalmol	13	7	616-21
Rhegmatogenous retinal detachment and uveitis.	Kerkhoff FT; Lamberts QJ; van den Biesen PR; Rothova A	2003	Ophthalmology	110	2	427-31
Factors related to severe uveitis at diagnosis in children with juvenile idiopathic arthritis in a screening program.	Chia A; Lee V; Graham EM; Edelsten C	2003	Am J Ophthalmol	135	6	757-62

The Ahmed drainage implant in the treatment of pediatric glaucoma.	Morad Y; Donaldson CE; Kim YM; Abdolell M; Levin AV	2003	Am J Ophthalmol	135	6	821-9
Patterns of uveitis in children presenting at a tertiary eye care centre in south India.	Narayana KM; Bora A; Biswas J	2003	Indian J Ophthalmol	51	2	129-32
Analysis of pediatric uveitis cases at a tertiary referral center.	Kump LI; Cervantes-CastaÃ±eda RA; Androudi SN; Foster CS	2005	Ophthalmology	112	7	1287-92
Outcomes of treatment with immunomodulatory therapy in patients with corticosteroid-resistant juvenile idiopathic arthritis-associated chronic iridocyclitis.	Yu EN; Meniconi ME; Tufail F; Baltatzis S; Foster CS	2005	Ocul Immunol Inflamm	13	5	353-60
Clinical course and outcome of uveitis in children.	Friling R; Kramer M; Snir M; Axer-Siegel R; Weinberger D; Mukamel M	2005	J AAPOS	9	4	379-82
Tumor necrosis factor-alpha blocker in treatment of juvenile idiopathic arthritis-associated uveitis refractory to second-line agents: results of a multinational survey.	Foeldvari I; Nielsen S; KÃ¼mmerle-Deschner J; Espada G; Horneff G; Bica B; Olivieri AN; Wierk A; Saurenmann RK	2007	J Rheumatol	34	5	1146-50
Course, complications, and outcome of juvenile arthritis-related uveitis	Sabri K; Saurenmann RK; Silverman ED; Levin AV	2008	J AAPOS	12	6	539-45
Biomarkers of chronic uveitis in juvenile idiopathic arthritis: predictive value of antihistone antibodies and antinuclear antibodies.	Nordal EB; Songstad NT; Berntson L; Moen T; Straume B; Rygg M	2009	J Rheumatol	36	8	1737-43
Ocular threat in juvenile idiopathic arthritis.	Marvillet I; Terrada C; Quartier P; Quoc EB; Bodaghi B; Prieur AM	2009	Joint Bone Spine	76	4	383-8
Epidemiology and course of disease in childhood uveitis.	Smith JA; Mackensen F; Sen HN; Leigh JF; Watkins AS; Pyatetsky D; Tessler HH; Nussenblatt RB; Rosenbaum JT; Reed GF; Vitale S; Smith JR; Goldstein DA	2009	Ophthalmology	116	8	1544-51
Outcomes of cataract surgery in children with chronic uveitis.	QuiÃ±ones K; Cervantes-CastaÃ±eda RA; Hynes AY; Daoud YJ; Foster CS	2009	J Cataract Refract Surg	35	4	725-31
Uveitis in childhood: an Italian clinical and epidemiological study.	Paroli MP; Spinucci G; Liverani M; Monte R; Pezzi PP	2009	Ocul Immunol Inflamm	17	4	238-42
Long-term follow-up of patients with uveitis associated with juvenile idiopathic arthritis: a cohort study	Skarin A; Elborgh R; Edlund E; Bengtsson-Stigmar E	2009	Ocul Immunol Inflamm	17	2	104-8

Risk factors for development of uveitis differ between girls and boys with juvenile idiopathic arthritis.	Saurenmann RK; Levin AV; Feldman BM; Laxer RM; Schneider R; Silverman ED	2010	Arthritis Rheum	62	6	1824-8
Male gender and poor visual outcome in uveitis associated with juvenile idiopathic arthritis.	Kalinina Ayuso V; Ten Cate HA; van der Does P; Rothova A; de Boer JH	2010	Am J Ophthalmol	149	6	987-93
Pars plana vitrectomy in the management of paediatric uveitis: the Massachusetts Eye Research and Surgery Institution experience.	Giuliari GP; Chang PY; Thakuria P; Hinkle DM; Foster CS	2010	Eye (Lond)	24	1	7-13
Timing of uveitis onset in oligoarticular juvenile idiopathic arthritis (JIA) is the main predictor of severe course uveitis.	Zannin ME; Buscain I; Vittadello F; Martini G; Alessio M; Orsoni JG; Breda L; Rigante D; Cimaz R; Zulian F	2012	Acta Ophthalmol	90	1	91-5
Risk factors associated with the relapse of uveitis in patients with juvenile idiopathic arthritis: a preliminary report	Saboo US; Metzinger JL; Radwan A; Arcinue C; Parikh R; Mohamed A; Foster CS	2013	J AAPOS	17	5	460-4
Risk factors for loss of visual acuity among patients with uveitis associated with juvenile idiopathic arthritis: the Systemic Immunosuppressive Therapy for Eye Diseases Study	Gregory AC 2nd; Kempen JH; Daniel E; Kaçmaz RO; Foster CS; Jabs DA; Levy-Clarke GA; Nussenblatt RB; Rosenbaum JT; Suhler EB; Thorne JE	2013	Ophthalmology	120	1	186-92
Immunomodulatory therapy with tumour necrosis factor Î± inhibitors in children with antinuclear antibody-associated chronic anterior uveitis: long-term results.	Doycheva D; Zierhut M; Blumenstock G; Stuebiger N; Januschowski K; Voykov B; Deuter C	2014	Br J Ophthalmol	98	4	523-8
Erythrocyte sedimentation rate as baseline predictor for the development of uveitis in children with juvenile idiopathic arthritis.	Haasnoot AJ; van Tent-Hoeve M; Wulffraat NM; Schalij-Delfos NE; Los LI; Armbrust W; Zuithoff NP; de Boer JH	2015	Am J Ophthalmol	159	2	372-7.e1
Development of macular edema and impact on visual acuity in uveitis associated with juvenile idiopathic arthritis.	de Boer J; Steijaert A; van den Bor R; Stellato R; Ossewaarde-van Norel J	2015	Ocul Immunol Inflamm	23	1	67-73
Temporal change in prevalence and complications of uveitis associated with juvenile idiopathic arthritis:data from a cross-sectional analysis of a prospective nationwide study	Tappeiner C; Klotsche J; Schenck S; Niewerth M; Minden K; Heiligenhaus A	2015	Clin Exp Rheumatol	33	6	936-44
Chronic Anterior Uveitis in Children	Couto C; Frick MM; LaMattina K; Schlaen A; Khoury M; Lopez MM; Hurtado E; Espada G	2016	Ocul Immunol Inflamm	24	4	392-6
Impact of Antiinflammatory Treatment on the Onset of Uveitis in Juvenile Idiopathic Arthritis: Longitudinal Analysis From a Nationwide Pediatric Rheumatology Database	Tappeiner C; Schenck S; Niewerth M; Heiligenhaus A; Minden K; Klotsche J	2016	Arthritis Care Res (Hoboken)	68	1	46-54
Incidence and predictors of Uveitis in juvenile idiopathic arthritis in a Nordic long-term cohort study.	Nordal E; Rypdal V; Christoffersen T; Aalto K; Berntson L; Fasth A; Herlin T; Nielsen S; Peltoniemi S; Straume B; Zak M; Rygg M	2017	Pediatr Rheumatol Online J	15	1	66

Risk Factors for the Development of Cataract in Children with Uveitis	Blum-Hareuveni T; Seguin-Greenstein S; Kramer M; Hareuveni G; Sharon Y; Friling R; Sharief L; Lightman S; Tomkins-Netzer O	2017	Am J Ophthalmol	177		139-143
The effect of anti-tumor necrosis factor alpha agents on the outcome in pediatric uveitis of diverse etiologies.	Deitch I; Amer R; Tomkins-Netzer O; Habot-Wilner Z; Friling R; Neumann R; Kramer M	2018	Graefes Arch Clin Exp Ophthalmol	256	4	801-808
Disease Remission in Children and Adolescents with Intermediate Uveitis: A Survival Analysis.	Borrego-Sanz L; Abásolo L; López-Abad C; Fernández-Gutiérrez B; García-Feijoo J; Diaz-Valle D; Pato E; Rodriguez-Rodriguez L	2018	Ophthalmologica	239	22	151-158
Risk markers of juvenile idiopathic arthritis-associated uveitis in the Childhood Arthritis and Rheumatology Research Alliance (CARRA) Registry	Angeles-Han ST; Pelajo CF; Vogler LB; Rouster-Stevens K; Kennedy C; Ponder L; McCracken C; Lopez-Benitez J; Drews-Botsch C; Prahalad S	2013	J Rheumatol	40	1	2088-96

Pubmed search: (uveitis) AND (cohort study) AND ((infant[MeSH] OR child[MeSH] OR adolescent[MeSH]))

Limited to publications from database inception to 2nd April 2018

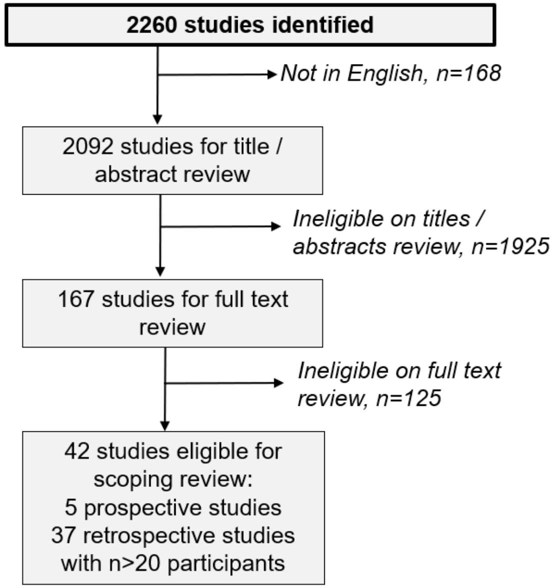

Supplement: Supplementary Document 1 — List of studies used to inform the long-list in Phase 1. [file Data_Sheet_1.PDF]
