# Supplementary material for: Development of a Nationally Agreed Core Clinical Dataset for Childhood Onset Uveitis
Source: Front Pediatr. 2022 Jun 21;10:881398. doi: 10.3389/fped.2022.881398 (PMC9253543; doi:10.3389/fped.2022.881398)
Supplement: Supplementary Document 2 — List of cohorts, registries and core outcome sets used to inform the long-list in Phase 1. [file Data_Sheet_2.PDF]

Supplementary document 2: List of cohorts, registries and core outcome sets used to inform the long-list in Phase 1

Organisation	Country / Countries	Study / registry	Reference for protocol / methods
National			
BSPAR [British Society of Paediatric and Adolescent Rheumatology]	UK	CAPS cohort [Childhood Arthritis Prospective Study]	Davies R, Carrasco R, Foster HE et al. Treatment prescribing patterns in patients with juvenile idiopathic arthritis (JIA): Analysis from the UK Childhood Arthritis Prospective Study (CAPS). Semin Arthritis Rheum. 2016 Oct;46(2):190-195. doi: 10.1016/j.semarthrit.2016.06.001. Epub 2016 Jun 8. PMID: 27422803; PMCID: PMC5052142.
BSPAR	UK	BCRD Registry [Biologics for Children with Rheumatic Diseases] and UK JIA Biologics Register	Kearsley-Fleet L, Davies R, Baildam E, et al. Factors associated with choice of biologic among children with Juvenile Idiopathic Arthritis: results from two UK paediatric biologic registers. Rheumatology (Oxford). 2016 Sep;55(9):1556-65. doi: 10.1093/rheumatology/kev429. Epub 2016 Jan 4. PMID: 26732349; PMCID: PMC4993954.
German Paediatric Rheumatology Network	Germany	ICON [Inception Cohort of Newly Diagnosed Children with JIA]	Sengler C, Klotsche J, Niewerth M, et al. The majority of newly diagnosed patients with juvenile idiopathic arthritis reach an inactive disease state within the first year of specialised care: data from a German inception cohort. RMD Open. 2015 Dec 8;1(1):e000074. doi: 10.1136/rmdopen-2015-000074. PMID: 26688748; PMCID: PMC4680591.
German Paediatric Rheumatology Network	Germany	NPRD [National Pediatric Rheumatology Database]	Minden K, Niewerth M, Listing J, et al. Health care provision in pediatric rheumatology in Germany--national rheumatologic database. J Rheumatol. 2002 Mar;29(3):622-8. PMID: 11908581.
German Paediatric Rheumatology Network	Germany	BiKeR [Biologics in Pediatric Rheumatology Cohort Study] (children then transition to adult study, JuMBO [Juvenile Arthritis Methotrexate Biologics Long-Term Observation Study])	Horneff G, De Bock F, Foeldvari I, et al. Safety and efficacy of combination of etanercept and methotrexate compared to treatment with etanercept only in patients with juvenile idiopathic arthritis (JIA): preliminary data from the German JIA Registry. Ann Rheum Dis. 2009 Apr;68(4):519-25. doi: 10.1136/ard.2007.087593. Epub 2008 Apr 15. PMID: 18413440.<br>Minden K, Niewerth M, Zink A, et al. Long-term outcome of patients with JIA treated wih etanercept, results of the biologic register JuMBO. Rheumatology

			(Oxford). 2012 Aug;51(8):1407-15. doi: 10.1093/rheumatology/kes019. Epub 2012 Mar 24. PMID: 22447885.
Pediatric Rheumatology Collaborative Study Group	Canada	ReACCh Out [Research in Arthritis in Canadian Children Emphasizing Outcomes Study]	Shiff NJ, Tucker LB, Guzman J, et al. Factors associated with a longer time to access pediatric rheumatologists in Canadian children with juvenile idiopathic arthritis. J Rheumatol. 2010 Nov;37(11):2415-21. doi: 10.3899/jrheum.100083. Epub 2010 Aug 17. PMID: 20716664.
Childhood Arthritis and Rheumatology Research Alliance	USA	CARRA [Childhood Arthritis and Rheumatology Research Alliance] Registry**	Beukelman T, Kimura Y, Ilowite NT, et al. The new Childhood Arthritis and Rheumatology Research Alliance (CARRA) registry: design, rationale, and characteristics of patients enrolled in the first 12 months. *Pediatr Rheumatol Online J*. 2017;15(1):30. Published 2017 Apr 17. doi:10.1186/s12969-017-0160-6
National Eye Institute (NEI)	USA	Systemic Immunosuppressive Treatment for Eye Diseases (SITE) Cohort Study**	Kempen JH, Daniel E, Gangaputra S, et al. Methods for identifying long-term adverse effects of treatment in patients with eye diseases: the Systemic Immunosuppressive Therapy for Eye Diseases (SITE) Cohort Study. Ophthalmic Epidemiol. 2008 Jan-Feb;15(1):47-55. doi: 10.1080/09286580701585892. PMID: 18300089.
Multinational			
Portuguese Society of Rheumatology	Brazil, Portugal, Madeira and Azores islands	Reuma.pt [Rheumatic Diseases Portuguese Registry]	Canhão H, Faustino A, Martins F, et al. Reuma.pt - the rheumatic diseases portuguese register. Acta Reumatol Port. 2011 Jan-Mar;36(1):45-56. PMID: 21483280.
Nordic Rheumatology network	Sweden, Finland, Denmark, Norway, Iceland	Nordic JIA Cohort**	Berntson L, Andersson Gäre B, Fasth A, et al. Incidence of juvenile idiopathic arthritis in the Nordic countries. A population based study with special reference to the validity of the ILAR and EULAR criteria. J Rheumatol. 2003 Oct;30(10):2275-82. PMID: 14528529.

Supplementary document 2: List of cohorts, registries and core outcome sets used to inform the long-list in Phase 1

PRINTO [Paediatric Rheumatology INternational Trials Organisation]	67 countries	PHARMACHILD (Prospective) [Pharmacovigilance in Juvenile Idiopathic Arthritis Patients]; The JIA Classification study; and the STARS Trial [the comparison of STep-up and step-down therapeutic strategies in childhood ARthritiS trial]	Swart J, Giancane G, Horneff G, Paediatric Rheumatology International Trials Organisation (PRINTO), BiKeR and the board of the Swedish Registry et al. Pharmacovigilance in juvenile idiopathic arthritis patients treated with biologic or synthetic drugs: combined data of more than 15,000 patients from Pharmachild and national registries. Arthritis Res Ther. 2018 Dec 27;20(1):285. doi: 10.1186/s13075-018-1780-z. PMID: 30587248; PMCID: PMC6307151.
MIWGUC [Multinational Interdisciplinary Working Group for Uveitis in Childhood]	Pan-European, UK, with proposed extension to involve USA	MIWGUC consensus on core outcomes for paediatric uveitis**	Heiligenhaus A, Foeldvari I, Edelsten C, Smith JR, Saurenmann RK, Bodaghi B, de Boer J, Graham E, Anton J, Kotaniemi K, Mackensen F, Minden K, Nielsen S, Rabinovich EC, Ramanan AV, Strand V; Multinational Interdisciplinary Working Group for Uveitis in Childhood. Proposed outcome measures for prospective clinical trials in juvenile idiopathic arthritis-associated uveitis: a consensus effort from the multinational interdisciplinary working group for uveitis in childhood. Arthritis Care Res (Hoboken). 2012 Sep;64(9):1365-72. doi: 10.1002/acr.21674. PMID: 22438273.

**Previously identified in rapid review (supplemental document 1)
