# Supplementary material for: Development of a Nationally Agreed Core Clinical Dataset for Childhood Onset Uveitis
Source: Front Pediatr. 2022 Jun 21;10:881398. doi: 10.3389/fped.2022.881398 (PMC9253543; doi:10.3389/fped.2022.881398)
Supplement: Supplementary Document 3 — Long list of candidate items. [file Data_Sheet_3.PDF]

Supplementary document 3: Long list of candidate data items

Data item	**Format of data entry**
Hospital number	Alphanumeric / numeric {necessary data item}
NHS number	Numerical (unique identifier) {necessary data item}
Surname	Free text {necessary data item}
Forename	Free text {necessary data item}
DOB	DD/MM/YYYY {necessary data item}
Gender	Male/Female/Other {necessary data item}
Ethnicity	UK Office National Statistics classification {necessary data item}
Postcode	Free text {necessary data item}
Mode detection problem	Selection from categories: School screening / Routine testing / Routine surveillance / Symptoms or concerns / Other - free text
Date problem started / detected	MM/YYYY
Referral source	Selection from categories: GP / Rheum / Optician / School screening / A&E / Secondary care / Tertiary care / Other - free text
Symptoms: asymptomatic	Yes / no / unsure
Symptom details	Selection from categories: No details/Asymptomatic/Blurred vision/Redness/Pain/discomfort/Change in eye appearance/Unknown/Other - free text
Other history	Free text
Other referral details	Free text
Date first seen	DD/MM/YYYY
Date uveitis first diagnosed	DD/MM/YYYY
Date topical treatment started	DD/MM/YYYY
Previous uveitis event	Selection from categories: None/Surgery/Treatment started/Treatment stopped/Increased IOP/VA loss/Other event - free text
Date previous uveitis event	DD/MM/YYYY
Details	Free text
Newly diagnosed / previously known systemic diagnoses	Selection from categories: None/ JIA (inc ILAR subtype)/ Definite Sarcoid/ Presumed sarcoid /Probable sarcoid {Definitions: https://bjo.bmj.com/content/103/10/1418} /Blau / Behcets/ TINU/ Psoriasis/ IBD/ Vasculitis/ Other (Free text)
If JIA, subtype	Limited to Systemic/Oligo/Poly/Psoriatic/ERA/Undifferentiated
If vasculitis, subtype	Free text
Date onset of systemic disease	Date/time
Details systemic diagnosis	Free text
Systemic review (where diagnosis unknown, or undifferentiated JIA)	Yes / no with selection from: fever/rash or spots/weight change/lymphadenopathy/lower GI symptoms/oral or other upper GI symptoms/respiratory symptoms/other with details

Supplementary document 3: Long list of candidate data items

Height (mg)	Numerical
Weight (kg)	Numerical
Family history	Selection from categories: No relevant history/Unknown/Glaucoma/Uveitis (Type)/JIA/Spondylopathy/RA/Sarcoid/SLE/TB/MS/Coeliac/Thyroid/IBD/Other autoimmune disease/Any other - free text
Details family history	Text
Number of joints with active arthritis	Numerical (from rheumatology team)
Physician's global assessment of systemic disease activity on a visual analogue scale	Numerical 0-100 (from rheumatology team)
Previous / current ocular meds	Selection from categories: Predforte / Dexamethasone / Cyclopentolate/ Other - free text
Date started	DD/MM/YYYY
Date stopped (if no longer in use)	DD/MM/YYYY
Previous / current systemic medication	Text
Date previous systemic medication started	DD/MM/YYYY
Date previous systemic medication stopped (if no longer in use)	DD/MM/YYYY
Date	DD/MM/YYYY
Chart used	Selection from categories: Standard LogMAR/Kays pictures/Cardiff cards/Other - free text
VA tested with	Limited to Unaided/Aided/Pinhole
VA recorded	Limited to LogMAR/Snellen/CPD/CF/HM/PL/NPL
Eye tested	Limited to RE/LE/BEO
Manifest deviation	Yes / No + detail
Colour vision	Not done / Normal/Abnormal, free text
Best corrected near vision	Not done / Normal/Abnormal, free text
Contrast vision	Not done / Normal/Abnormal, free text
RAPD	Yes/No
Any change to anterior segment	Yes/No
Any change to posterior segment	Yes/No
AC cells pre dilation	Limited to 0/0.5+/1+/2+/3+/4+ {Definitions https://www.sciencedirect.com/science/article/pii/S0002939405004071?via%3Dihub}
AC flare pre dilation	Limited to 0/0.5+/1+/2+/3+/4+ {Definitions https://www.sciencedirect.com/science/article/pii/S0002939405004071?via%3Dihub}

Supplementary document 3: Long list of candidate data items

AC Laser flare photometry	Not done / Done + photon units
Keratic precipitates	No / Yes + descriptor
Lens	Clear/Cataract/Aphakic/Pseudophakic/ Other free text
If cataract: Predominant cataract type	Limited to: Total / Cortical / Anterior / Posterior / Nuclear
Band keratopathy	Yes - No/yes peripheral/yes central axis
Pupillary synechiae	No / Yes + degree (in clock hours)
Iris bombe	Yes/No
Iris hyperaemia	Yes/No
Other iris abnormality details	PAS (in degrees)/Nodules/Atrophy/Other free text
Pupillary membrane formation	Yes/No
Other anterior segment	Selection from categories: None/Scleritis/Keratitis/Iris atrophy/Other: specify - free text
Dilation	No / yes
Posterior segment healthy	Yes/No/No view
Vitreous haze	Limited to: 0/0.5+/1+/2+/3+/4+ {Definitions https://www.sciencedirect.com/science/article/pii/S0002939405004071?via%3Dihub}
Vitreous cells	Yes/No
Vitreous opacities	Selection from categories: None/ Exudate over pars plana - snowbanking/Snowballs/ Other - free text
CD ratio	0 - 1 Decimal
Disc swelling	Yes/No
Other disc changes	Selection from categories: None / Disc haemorrhages / Hyperaemia / Vessel engorgement / Vessel obscuration / Other - free text
Optic atrophy	Yes/No
ERM	Yes/No
Macula oedema present	No/Yes-clinical/Yes-OCT
Retinal vasculitis	No/Yes
Any active chorioretinal lesion	No/Peripheral only/Macula/Not done
Other posterior segment	Selection from categories: None/ Retinal neovascularisation / CNV / Subretinal fluid / Subretinal mass / Vitreous haemorrhage/Other structural macula change/Retinal detachment/Other peripheral retinal change, free text
IOP (mmHg)	Numerical
Test used IOP	Goldmann/I-care/Digital
Glaucoma	Yes/No
CCT	Numerical

Supplementary document 3: Long list of candidate data items

Type of uveitis	Limited to Anterior/Intermediate/Posterior/Pan {Definitions https://www.sciencedirect.com/science/article/pii/S0002939405004071?via%3Dihub}
Subtype of uveitis	Limited to: Iritis / Iridocyclitis / Anterior cyclitis / Pars planitis / Posterior cyclitis / Hyalitis / Focal, multifocal, or diffuse choroiditis / Chorioretinitis / Retinochoroiditis / Retinitis / Neuroretinitis {Definitions https://www.sciencedirect.com/science/article/pii/S0002939405004071?via%3Dihub}
Cause of reduced VA	Selection from categories: Cataract/Refractive error/Vitreous/CMO/Amblyopia/AC inflammation/Cornea/CNV / Other:specify - free text
Topical corticosteroid drop	Selection from categories: None/Dexamethasone/Predforte/Lotemax/Maxitrol/FML/Other - free text
Steroid drops daily frequency	Selection from categories: alt day/1/2/3/4/6/2 hourly/hourly/other - free text
Glaucoma drops	Selection from categories {generic names used}: None/Timolol 0.25bd/Timolol 0.5LA/Trusopt/Azopt/Cosopt/Azarga/Alphagan/Iopidine/Latanoprost/Bimatoprost/Travoprost/Xalacom/Ganfort/Other - free text
Mydriatic drops	Selection from categories: None/Cyclopentolate/Tropicamide/Atropine/Other - free text
Mydriatic drop daily use frequency	Selection from categories: alt days/1/2/3/4
New / changed systemic treatment	Yes/No
Date started	DD/MM/YYYY (for each individual treatment)
Date stopped	DD/MM/YYYY (for each individual treatment)
Drug / route / dose	Free text, or choices as in rows below:
Prednisolone	Numerical
Prednisolone tapering regimen	Text
Methotrexate (mg)	Numerical
Methotrexate route	SC / PO
MMF (mg)	Numerical
Adalimumab	Yes/No
Infliximab	Yes/No
Infliximab interval (weeks)	Numerical
Tocilizumab	Yes/No
Tocilizumab interval (weeks)	Numerical
Diamox (mg)	Numerical
Other	Free text
Fundal photography	Not done/ Done + Normal/Done + Abnormal
OCT macula	Not done/ Done + Normal +CMT/Done + Abnormal+findings+CMT
OCT optic nerve	Not done/ Done + Normal/Done + Abnormal

Supplementary document 3: Long list of candidate data items

FFA	Not done/ Done + Normal/Done + Abnormal
ICG	Not done/ Done + Normal/Done + Abnormal
Other imaging	Free text with imaging type and result
ANA	Not done/ Done
HLA-B27	Not done/ Done
ESR	Not done/ Done
CRP	Not done/ Done
ACE	Not done/ Done
IgA/E/M/G	Not done/ Done
ASO titre	Not done/ Done
FBC	Not done/ Done
ANCA	Not done/ Done
ACA	Not done/ Done
ENA	Not done/ Done
NOD2 test	Not done/ Done + Negative / Done + known mutation / Done + other mutation
Other Genetic tests (including HLA mrkers)	Not done/ Done + Negative / Done + uncertain pathogenicity / Done + diagnosis made
Liver function test	Not done/ Done
Renal function	Not done/ Done
Rheumatoid factor	Not done/ Done
QTB	Not done/ Done
CXR	Not done/ Done + Normal/Done + Abnormal
Vitamin D	Not done/ Done
Other	Free text
Other notable positive laboratory findings	No / Yes, specify free text
Referral to paediatrician	Yes/No
Referral to paediatric rheumatologist	Yes/No
Referral to other ophthalmologist	Yes/No
Other referral	Free text
Clinical judgment on response to last therapeutic change	Limited to: Inactive / Unchanged / Worsening activity / Improved activity / Unchanged / Remission {Definitions https://www.sciencedirect.com/science/article/pii/S0002939405004071?via%3Dihub}
Drug monitoring blood tests	Not required / Hospital /GP /Paediatrician
CNS involved in care	Yes / No

Supplementary document 3: Long list of candidate data items

Reason treatment stopped (if stopped)	eg, Remission/inefficacy/side effects/unknown
Serious adverse drug response	Free text
Date of any surgery	DD/MM/YYYY
Indication for surgery	Selection from categories: Intractable inflammation / Iris bombe / Cataract / Glaucoma / BK / RD / ERM / Other - free text
Type of surgery	Selection from categories: Intraocular steroid/Periocular steroid/Lens extraction / IOL /YAG capsulotomy/ Surgical caps + Vity/ PI/RD surgery/Dexamethasone implant (Ozurdex)/Intravitreal anti-VEGF/Vitrectomy/Glaucoma shunt surgery/Glaucoma trabeculectomy/Glaucoma cyclodiode/Removal BK/Other - free text
Peri-operative steroid pulse	No / Yes, details - free text
Other relevant surgical details	Free text
Predicted post-op VA (LogMAR)	Same / better by >2 lines
Complications following surgery	Yes/No
Complication details	Text
